# Cellular and Subcellular Phosphate Transport Machinery in Plants

**DOI:** 10.3390/ijms19071914

**Published:** 2018-06-29

**Authors:** Sudhakar Srivastava, Munish Kumar Upadhyay, Ashish Kumar Srivastava, Mostafa Abdelrahman, Penna Suprasanna, Lam-Son Phan Tran

**Affiliations:** 1 Institute of Environment and Sustainable Development, Banaras Hindu University, Varanasi 221005, UP, India; sudhakar.srivastava@gmail.com (S.S.); munish007up@gmail.com (M.K.U.); 2 Plant Stress Physiology and Biotechnology Section, Nuclear Agriculture & Biotechnology Division, Bhabha Atomic Research Centre, Mumbai 400085, India; ashishbarc@gmail.com (A.K.S.); penna888@yahoo.com (P.S.); 3 Arid Land Research Center, Tottori University, 1390 Hamaska, Tottori 680-0001, Japan; meettoo2000@tottori-u.ac.jp; 4 Botany Department, Faculty of Sciences, Aswan University, Aswan 81528, Egypt; 5 Plant Stress Research Group & Faculty of Applied Sciences, Ton Duc Thang University, Ho Chi Minh City 700000, Vietnam; 6 Stress Adaptation Research Unit, RIKEN Center for Sustainable Resource Science, Yokohama 230-0045, Kanagawa, Japan

**Keywords:** phosphate, subcellular organelles, transporters

## Abstract

Phosphorus (P) is an essential element required for incorporation into several biomolecules and for various biological functions; it is, therefore, vital for optimal growth and development of plants. The extensive research on identifying the processes underlying the uptake, transport, and homeostasis of phosphate (Pi) in various plant organs yielded valuable information. The transport of Pi occurs from the soil into root epidermal cells, followed by loading into the root xylem vessels for distribution into other plant organs. Under conditions of Pi deficiency, Pi is also translocated from the shoot to the root via the phloem. Vacuoles act as a storage pool for extra Pi, enabling its delivery to the cytosol, a process which plays an important role in the homeostatic control of cytoplasmic Pi levels. In mitochondria and chloroplasts, Pi homeostasis regulates ATP synthase activity to maintain optimal ATP levels. Additionally, the endoplasmic reticulum functions to direct Pi transporters and Pi toward various locations. The intracellular membrane potential and pH in the subcellular organelles could also play an important role in the kinetics of Pi transport. The presented review provides an overview of Pi transport mechanisms in subcellular organelles, and also discusses how they affect Pi balancing at cellular, tissue, and whole-plant levels.

## 1. Introduction

**Figure 1:**
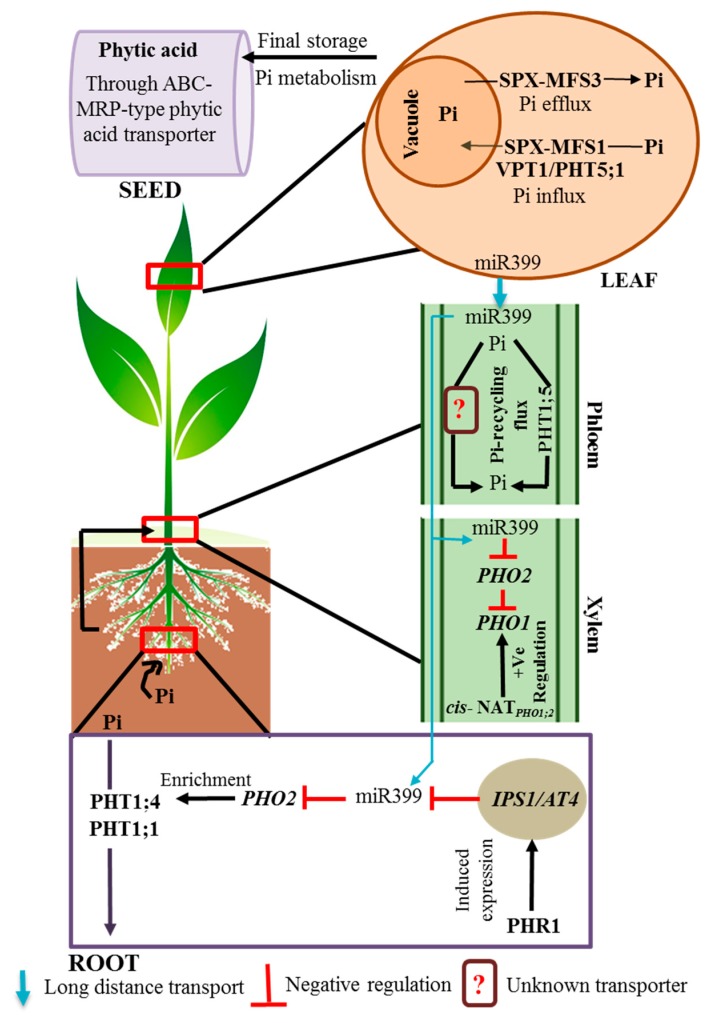
Overview of phosphate (Pi) transport in *Arabidopsis* plants. The transport route is shown in four parts: uptake from soil to roots, transport from roots to shoots, unloading in shoots and subcellular organelles, and transport to seeds in the form of phytic acid. The high-affinity Pi (PHT1) family (PHT1;1 and PHT1;4) of transporters plays a major role in Pi uptake from soil to roots. The PHO1 protein increases root-Pi xylem loading, whereas PHT1;5 plays a key role in the retranslocation of Pi from shoots to roots, and Pi mobilization to reproductive organs. In plant cell, vacuoles act as the primary intracellular compartments for Pi storage, and SPX-MFS1 and SPX-MFS3/PHT5;1 mediate vacuolar Pi influx and efflux, respectively. Furthermore, Pi is metabolized and transported from leaves to seeds in the form of phytic acid by the ABC-MRP-type phytic acid transporter. The levels of PHT1, PHO1 and PHO2 transporters are regulated by miR399 and *cis*-NAT*_PHO_*_1;2_ in xylem, and by miR399 and *IPS1/AT4* in roots. ABC-MRP, ATP binding cassette-multidrug resistance-associated protein; *AT4*, *Arabidopsis thaliana 4*; *IPS1*, induced by phosphate starvation 1; *cis*-NAT*_PHO_*_1;2_, *cis*-natural antisense transcript phosphate transporter 1;2; PHT, high-affinity phosphate transporter; PHR1, phosphate starvation response 1; Pi, phosphate; PHO1, phosphate transporter 1; SPX-MFS3, SYG1/PHO81/XPR1 major facility superfamily 3; SPX-MFS1, SPX major facilitator superfamily 1; VPT1, vacuolar phosphate transporter 1.

Phosphorus (P) is an essential macronutrient for sustaining optimal plant growth, and plays a significant role in a diverse array of cellular processes, including energy production, redox reactions, photosynthesis, and phosphorylation/dephosphorylation-based reactions [1,2]. Soil-P exists in various organic and inorganic forms due to the decomposition of soil organic matter and weathering of parent rock materials [3]. However, the various P forms differ in their behavior in soils depending on the soil’s pH and the size of mineral particles [3,4]. Among several soil-P forms, inorganic phosphate (PO_4_^3−^, H_2_PO_4_^−^, and HPO_4_^2−^, referred to as Pi hereafter) is the most readily accessible for plants; however, bioavailable Pi concentrations rarely exceed 1–10 µm in soil solutions [5,6,7]. In acidic soils, Pi is mostly adsorbed onto the surface of iron (Fe)/aluminum (Al) oxides, such as hematite, gibbsite, and goethite, due to ionic strength, thus trapping Pi in soil nanopores, thereby becoming unavailable to plants [3,4]. In calcareous-to-neutral soils, Pi can be precipitated with calcium (Ca^2+^), generating a dicalcium phosphate form that is available to plants; however, dicalcium phosphate can also be transformed into more stable forms, such as hydroxyapatite and octocalcium phosphate, which are less available to plants under alkaline soil-pH conditions [3]. To maintain crop productivity, farmers use enormous amounts of Pi-fertilizers derived from non-renewable rock phosphate [5,6,7]. However, the increasing demand for high crop yields due to the ever-increasing world population combined with the gradual decrease of global P reserves requires us to address how plants uptake, transport, and store Pi under conditions of limited Pi availability [5,6,7]. Plants have evolved several morphological, physiological, and molecular changes, including changes in root architecture, secretion of organic acids and acid phosphatases, accumulation of anthocyanin pigments, and improved Pi uptake efficiency to sustain their growth under Pi-deficient conditions [4]. Plants have developed specialized uptake/transport system at the root/soil interface for efficient Pi uptake from the soil and for transporting Pi across intracellular compartments [6]. In the event of Pi deficiency, one of the early responses includes lowering cytosolic Pi levels, resulting in transcriptional reprogramming, as well as Pi redistribution [8]. Plants are equipped with both low- and high-affinity Pi transporters that mediate Pi uptake and root-to-shoot transport [9,10] (Figure 1). Furthermore, dedicated sets of transporters are known to facilitate Pi redistribution at tissue, cellular, and subcellular levels [3,6,10].

Since the Pi concentration in soil solution is very low (<10 µM, as written above), while Pi concentration inside the plant cell is much higher (1–10 mM), root cells need to absorb Pi against the steep concentration gradient occurring between the cytosol and the soil solution [11,12]. Additionally, H_2_PO_4_^−^ and HPO_4_^2−^ anions need to overcome the negative membrane potential to enter the plant cell, which requires an energized transport system via H^+^/Pi co-transport [11,12]. Therefore, Pi does not enter the cell as H_2_PO_4_^−^ or HPO_4_^2−^ only; instead, it is accompanied by other cations like H^+^ to avoid membrane hyperpolarization [11,12]. Indeed, investigations during Pi uptake demonstrated decreased cytoplasmic pH in a *Catharanthus roseus* cell suspension, but increased pH in the extracellular medium [13], as well as the acidification of cytoplasmic pH in the root hairs of *Limmobium stoloniferum* [14]. After Pi uptake into the root symplasm, Pi can go via different routes: (i) Pi enters the cell cytoplasm (metabolic pool), where the primary access form of Pi into organic molecules occurs via anhydride bond formation as the γ-phosphate group of ATP; (ii) Pi (H_2_PO_4_^−^ or HPO_4_^2−^) is secreted into the xylem for long-distance translocation to aerial parts of the plant; and (iii) Pi is stored in vacuoles for the maintenance of Pi homeostasis [11,12]. Additionally, Pi transport from the phloem to the xylem primarily occurs in the form of H_2_PO_4_^−^ or HPO_4_^2−^; however, organic Pi compounds such as hexose-phosphates and ATP are also detectable in phloem sap [11,12].

Significant progress was made in understanding Pi transport and utilization mechanisms, which are more or less conserved across the plant kingdom [5,6,15]. A few plant species have a unique ability to tackle adverse effects of Pi deficiency. For instance, *Hakea prostrate* from the Proteaceae family evolved in severely Pi-deficient soils of southwestern Australia; thus, *H. prostrate* is highly efficient in managing Pi deficiency as it possesses some unique features [16]. These include cluster roots for efficient Pi uptake, delayed greening, altered Pi allocation to ribosomes, changes in membrane lipid composition, highly efficient photosynthetic Pi use, efficient remobilization of Pi from older senescing leaves, and high-Pi-containing seeds for the initiation of life in Pi-deficient environments [16]. This observation suggests that subcellular Pi transport and its reallocation into various plant parts are important factors for maintaining plant growth under both Pi-repleted and Pi-depleted conditions. Recently, with progress in functional genomics, the roles of novel genes associated with subcellular Pi transport and regulation were investigated. The pH of the cytoplasm and subcellular compartments, as well as the intracellular membrane potential, influences Pi transport at the subcellular level. While pH affects the chemical species of Pi, membrane potentials determine the feasibility of Pi import/export [15] (Table 1).

In view of this background information, the presented paper reviews our current understanding, gained mainly in *Arabidopsis thaliana* and rice (*Oryza sativa*), to provide an integrated view of Pi uptake from the soil and its transport mechanisms in/among various organs and subcellular organelles. Future research directions are discussed to develop suitable strategies for the development of crops better suited for growing under Pi-deficient conditions.

## 2. Phosphate Transporters and Their Role in Pi Acquisition, Translocation, and Remobilization in Various Organs

The transport of Pi across cell membranes is a critical stage in the regulation of Pi use. During the past few years, several transporter genes that mediate Pi transport processes were identified (Figure 1). However, the functions of many Pi transporters still remain elusive. *Arabidopsis* and rice plants contain five high-affinity Pi transporter (PHT1, PHT2, PHT3, PHT4, and PHT5) families that are distinguished based on their protein sequences, locations, and functions [23]. Table 2 summarizes known transporters for uptake at the root surface, root-to-shoot translocation, and the unloading of Pi in shoots.

The PHT1 proteins are plasma membrane (PM) proton-coupled Pi-symporters that mediate Pi acquisition from the soil [23,27]. The *PHT1* gene family has more members than other *PHT* families. For example, the *Arabidopsis* (At) *AtPHT1* gene family contains nine Pi transporters (*AtPHT1;1* to *AtPHT1;9*), among which *AtPHT1;1* to *AtPHT1;4* are mainly involved in Pi uptake from the soil to roots [9]. Gene expression analysis in *Arabidopsis* showed that *AtPHT1;1*, *AtPHT1;2*, *AtPHT1;3*, and *AtPHT1;4* are mainly expressed in various types of root cells, suggesting that these transporters have similar and partially overlapping functions [36,39]. For example, AtPHT1;1 plays a major role when Pi supply is high, whereas AtPHT1;4 becomes predominant under Pi-deficient conditions in *Arabidopsis* [39]. Ayadi et al. [39] demonstrated that the *Arabidopsis atpht1;1*/*atpht1;2*/*atpht1;3*/*atpht1;4* quadruple and the *phosphate transporter traffic facilitator1*(*phf1*)/*atpht1;4* double mutants are impaired in Pi uptake from soil to roots. In addition, Wang et al. [27] reported that *WRKY45* is a positive regulator of *AtPHT1;1*, and the *Arabidopsis* transgenic *WRKY45*-over-expressing lines showed induced *AtPHT1;1* expression and increased Pi uptake. By contrast, the *atpht1;1* mutants showed decreased Pi uptake in comparison with wild-type (WT) plants. In addition to the four major transporters (AtPHT1;1 to AtPHT1;4), the roles of other AtPHT1 proteins in Pi acquisition and transport cannot be neglected. For example, Nagarajan et al. [24] demonstrated that the *Arabidopsis* AtPHT1;5 plays a significant role in Pi translocation from source to sink organs. The authors reported that under low-Pi conditions, *Arabidopsis atpht1;5-1* mutants exhibited a significant decline in Pi translocation into the shoots, and induced expression of several Pi starvation-response genes [e.g., *At4*, *digalactosyldiacylglycerol synthase 1* (*DGD1*), and *UDP-sulfoquinovose synthase 1* (*SQD1*)] [24]. However, under Pi-sufficient conditions, the *Arabidopsis atpht1;5-1* mutants had higher shoot-Pi content than WT, suggesting that AtPHT1;5 plays a significant role in Pi homeostasis between the source and sink organs, in accordance with the Pi states and developmental cues [24]. In *Arabidopsis*, AtPHT1;6 and AtPHT1;7 regulate Pi translocation from leaves to pollens under Pi-sufficient conditions, while AtPHT1;8 and AtPHT1;9 control Pi translocation from roots to shoots, but not from the soil to roots, especially under Pi-deficient conditions [9,24,36].

The *Arabidopsis* *phosphate transporter 1* (*PHO1*) gene family is another important Pi-transporter family, playing an essential role in long-distance Pi transport from roots to shoots, and is responsible for the regulation of Pi export from root epidermal and cortical cells into xylem vessels [40,41] (Figure 1). In *Arabidopsis*, *AtPHO1* is mainly expressed in the lower part of the hypocotyl and the stellar cells of the roots, which is in agreement with its role in Pi transport to the xylem. It was reported that the *atpho1* mutants were deficient in loading Pi from root epidermal cells into the xylem vessels, resulting in Pi deficiency in the shoots [40,42]. Among several *AtPHO1* homologs in *Arabidopsis*, only *AtPHO1* and *AtPHO1;H1* could recover the defects of the *atpho1* mutation, demonstrating that only *AtPHO1* and *AtPHO1;H1* are implicated in long-distance Pi transport from roots to shoots [32]. Recently, *AtPHO1;H3* was shown to be involved in the suppression of root-to-shoot Pi transport under zinc-deficient conditions in *Arabidopsis* [33], while the roles of remaining transporter genes, *AtPHO1;H2*, and from *AtPHO1;H4* to *AtPHO1;H10*, still need to be identified. In rice (Os), all three *PHO1* (*OsPHO1;1*, *OsPHO1;2*, and *OsPHO1;3*) genes possess a *cis*-natural antisense transcript positioned at the 5′ end of the genes, and only *OsPHO1;2* is highly induced in the roots under Pi-deficient conditions [43]. Characterization of the *ospho1;1* and *ospho1;2* single mutants indicated that only *ospho1;2* mutants had a significant decrease in Pi transport from roots to shoots, which was accompanied by high root-Pi and low shoot-Pi contents [43].

The rice OsPHT1 family comprises 13 Pi transporters [28], and with the exception of *OsPHT1;3*, *5*, *7*, and *12*, all other *OsPHT1* genes were studied in detail. Sun et al. [44] demonstrated that *OsPHT1;1* was expressed in various shoot and root cells under Pi-sufficient conditions, and transgenic rice plants overexpressing *OsPHT1;1* exhibited higher shoot-Pi content than WT, suggesting that OsPHT1;1 has a crucial role in Pi uptake and translocation under Pi-sufficient conditions. On the other hand, the low-affinity *OsPHT1;2* and high-affinity *OsPHT1;6* are strongly upregulated under Pi-deficient conditions in rice roots and shoots [35]. The authors suggested that OsPHT1;6 plays a broad role in Pi uptake from soil to roots, and probably in Pi translocation throughout the plant, whereas OsPHT1;2 mediates only Pi transport from roots to shoots [34]. *OsPHT1;4* is expressed in roots, leaves, ligules, stamens, and caryopses, and is induced under long-term Pi-deficient conditions [28]. Transgenic *OsPHT1;4*-overexpressing rice plants displayed a significant increase in Pi contents in roots and shoots, whereas *ospht1;4* mutants exhibited a decrease in Pi contents in the respective organs [28,29]. In addition, a gradual increase in the relative expression of *OsPHT1;4* was observed in the embryos from 10 to 20 days after pollination when compared with that in the panicle axis and endosperm, suggesting a potential role of OsPHT1;4 in Pi acquisition during embryogenesis [29]. In rice, OsPHT1;4 is also implicated in Pi remobilization from flag leaves to panicles, while OsPHT1;6 and OsPHT1;8 are involved in Pi remobilization from senescing leaves to young ones and grains [28,45,46]. Additionally, using ^33^Pi as a radiotracer, plausible involvement of OsPHT1;4, OsPHT1;6 and OsPHT1;8 in root-to-shoot Pi transport was also demonstrated [29,45,47]. A recent study by Jia et al. [47] reported that the transgenic rice *OsPHT1;8*-overexpressing lines exhibited a significant increase in both root- and shoot-Pi contents; however, the transgenic plants showed toxicity symptoms with a decrease in root and shoot biomass under highly Pi-supplied conditions. However, under limited-Pi conditions, *OsPHT1;8*-overexpressing lines exhibited normal root and shoot biomass similar to that of WT plants [47]. Thus, appropriate regulation of cytoplasmic Pi and/or organelle Pi levels is important for maintaining plant fitness under Pi-deficient conditions.

## 3. Subcellular Pi Transport and Balancing

Table 3 summarizes transporters involved in subcellular Pi transport. The Pi transport in various subcellular organelles is discussed below under separate subheadings.

### 3.1. Vacuole: The Pi Storehouse

**Figure 2:**
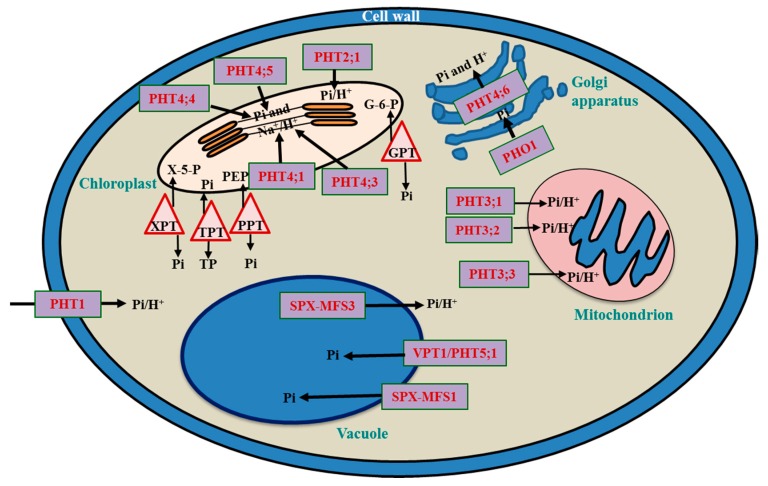
Subcellular localization of phosphate (Pi) transporters and translocators in *Arabidopsis* cells. PHT1, PHT2, PHT3, and SPX-MFS3 are proton-coupled Pi transporters. VPT1/PHT5;1 and SPX-MFS1 function as Pi channels for Pi influx from the cytoplasm into the vacuoles, whereas SPX-MFS3 mediates Pi efflux from the vacuoles into cytoplasm. PHT4 proteins mediate Na^+^/H^+^-dependent Pi transport from the cytosol to the chloroplasts or Golgi. PHO1 localizes to Golgi membranes and mediates Pi transport without an H^+^ gradient across the membrane. Among the PHT2 family members, only PHT2.1 was functionally characterized as a Pi importer in the chloroplast envelope. *AtPHT3* genes encode a small family of mitochondrial Pi transporters. These translocators mediate Pi transport in the exchange of various substrates. GPT, glucose 6-phosphate (G-6-P)/(Pi) translocator; PPT, phosphoenolpyruvate (PEP)/Pi translocator; TPT, triose phosphate (TP)/Pi translocator; XPT, xylulose-5-phosphate (X-5-P)/Pi translocator. PHT, high-affinity phosphate transporter; SPX-MFS3, SYG1/PHO81/XPR1 major facility superfamily 3; SPX-MFS1, SPX major facilitator superfamily 1; VPT1, vacuolar phosphate transporter 1; Black arrows show the influx and efflux of Pi in the vacuole, mitochondrion, Golgi apparatus, and chloroplast through the regulation of various PHT members.

Vacuoles are considered as the storehouse of Pi in plant cells. Under sufficiently Pi-supplied conditions, 70–95% of the intracellular Pi is stored inside the vacuoles in the form of orthophosphate in vegetative cells, whereas in plant seeds, Pi is stored in specialized protein storage vacuoles in the phytate form [7]. Since Pi may not be available at optimal concentrations during the entire course of the plant’s life cycle, excess Pi is taken up and stored in vacuoles under the conditions of high Pi availability [1]. Whenever there is a decline in Pi concentration in the cytosol, Pi supplies are operated by the vacuole-Pi pool [1]. Hence, optimized Pi influx and efflux from vacuoles are essential for maintaining Pi homeostasis in other organelles, tissues, and also at the whole-plant level. Recently, Liu et al. [1] reported the functional characterization of the *Arabidopsis* vacuolar phosphate transporter 1 (AtVPT1), an SYG1/PHO81/XPR1 (SPX) major facilitator superfamily3 (MFS3) domain protein, that mediates Pi influx from the cytoplasm into the vacuoles (Figure 2). Transgenic *Arabidopsis atvpt1* mutant plants were not able to retain more Pi content in the vacuoles than WT plants under high-Pi conditions, suggesting that disruption of *AtVPT1* reduced the adaptability of plants to the changed Pi availability [1]. In addition, *AtVPT1* expression was detected in younger tissues under normal conditions, but the expression level increased drastically in older leaves under highly Pi-supplied conditions, suggesting that AtVPT1 functions in detoxification resulting from high Pi levels in older tissues [1]. Liu et al. [48] identified a vacuolar transporter, named AtPHT5;1, in *Arabidopsis* via analysis of Pi translocation across the tonoplast using ^31^P-magnetic resonance spectroscopy. The authors found that AtPHT5;1 mediated Pi influx from the cytoplasm into vacuoles, and the *AtPHT5;1*-overexpressing lines accumulated more Pi in the vacuoles, leading to a decreased cytoplasmic-Pi level under Pi-repleted conditions (Table 3). Liu et al. [48] also heterologously expressed an *AtPHT5;1* homolog, the rice *OsSPX-MFS1* gene in yeast, and reported that OsSPX-MFS1 localized in the tonoplast of yeast cells, and mediated Pi influx into yeast vacuoles. By contrast, the transgenic rice *OsSPX-MFS3-*overexpressing lines exhibited a decrease in vacuole-Pi content, suggesting that OsSPX-MFS3 acts as a vacuolar low-affinity Pi transporter involved in Pi efflux from the vacuoles [19] (Figure 2).

The storage capacity for minerals in the vacuoles of various leaf cell types is compositionally different, and the mechanisms and reasons underpinning this compartmentalization are still elusive. For instance, in *Arabidopsis*, potassium (K^+^) and Pi accumulate preferentially in the vacuoles of bundle sheath and epidermal cells, whereas magnesium (Mg^2+^) and Ca^2+^ are stored at high concentrations (>60 mM) only in the vacuoles of mesophyll cells [49,50]. In addition, Ca^2+^ and Pi do not co-localize at high concentrations in the same vacuoles, as this results in insoluble calcium phosphate complexes [49,50]. However, an opposite trend was observed in cereal monocots, including *Sorghum bicolor* and wheat (*Triticum aestivum*), in which Ca^2+^ accumulated in the epidermal cells, whereas K^+^ and Pi accumulated in the mesophyll or bundle sheath cells [49,50]. Therefore, the cellular location of a particular mineral element is vigorous within an individual plant; however, the cell type that accumulates a given element can vary among plant species. The Ca^2+^/H^+^-antiporter (*CAX1*) was reported as a key regulator for cell-specific storage of Ca^2+^ to optimize transpiration, cell-wall expansion, and plant productivity [49,50]; however, identification of the mechanisms involved in the cell-specific storage of Pi is a future task.

### 3.2. Chloroplast

The chloroplast is an important organelle which generates ATP, the prime source of energy for multiple reactions inside the cell, through photophosphorylation [60]. For efficient ATP synthesis, the reactants (ADP and Pi) must be transported into the chloroplasts, while the product (ATP) must be simultaneously exported out of the chloroplasts to avoid any feedback inhibition [18]. In *Arabidopsis*, a total of 16 *plastidic phosphate translocator* (*pPT*) genes are present, including those encoding the triose phosphate (TP)/phosphate translocator (TPT), the phosphoenolpyruvate (PEP)/Pi translocator (PPT), the xylulose-5-phosphate/phosphate translocator (XPT), and the glucose-6-phosphate/phosphate translocator (GPT) proteins [61] (Figure 2). In addition, some truncated versions of PPTs, GPTs, and phosphate translocator homologs (PThs) were also detected [61]. In *Arabidopsis*, two plastidic ADP/ATP translocator (AATP1) and H^+/^Pi symporter (AtPHT2;1) were also identified and studied in detail [51,62] (Table 3). In order to maintain optimal rates of photosynthesis, triose-Pi is exported from chloroplast stroma to the cytosol by TPT in exchange for cytosolic Pi [63]. However, this transport activity depends on Pi availability, which is released during sucrose synthesis from the triose-Pi [63]. During the daytime, when sucrose synthesis slows down, Pi levels are reduced, leading to lower TPT activity and the diversion of triose-Pi into starch synthesis [64]. In addition, Pi release also maintains photosynthetic carbon assimilation [64]. Furthermore, if the sucrose biosynthesis pathway is impaired, Pi availability is reduced, leading to reduced ATP synthesis as found in the rice *tpt* mutants [63]. In addition to the TPT proteins, PPTs that mediate PEP/Pi exchange are present in plastid inner envelope membranes, and their encoding genes are exclusively expressed in both photosynthetic and heterotrophic tissues in *Arabidopsis* [61]. Since Pi transport in chloroplasts is tightly linked to the import and export of other metabolic intermediates, chloroplast-Pi homeostasis might play a role not only in maintaining ATP synthesis, but also in overall plant metabolism [61].

In *Arabidopsis*, members of the *AtPHT2* and *AtPHT4* families are expressed in chloroplasts [60]. Of the *AtPHT2* family, *AtPHT2;1* was found to be expressed predominantly in green tissues, and localize on the chloroplast inner envelope membrane [51]. Furthermore, *Arabidopsis atpht2;1* mutants were shown to lack the ability to transport Pi inside the chloroplasts [51] (Table 3). Guo et al. [52] analyzed five members of the AtPHT4 family in *Arabidopsis*, and found that AtPHT4;1 and AtPHT4;4 are localized in leaf chloroplasts, AtPHT4;3 and AtPHT4;5 in shoot plastids, and AtPHT4;2 in root plastids. All AtPHT4 proteins were found to mediate H^+^-dependent Pi transport; however, the direction of the Pi transport was not conclusively demonstrated [65]. On the basis of the transport activity of expressed proteins in yeast, Guo et al. [65] suggested that AtPHT4 proteins might be involved in Pi export from plastids toward the cytoplasm. Irigoyen et al. [53] studied AtPHT4;2, and suggested that it may function in Pi export from plastids. The *atpht4;1* mutants have growth defects which are associated with reduced Pi levels in stroma and decreased ATP synthase activity in chloroplasts [66] (Figure 2).

### 3.3. Mitochondria

In addition to chloroplasts, mitochondria also generate ATP through oxidative phosphorylation. Pi is imported into mitochondria through a Pi carrier (PiC), while ATP is exported from mitochondria via an ADP/ATP carrier (AAC) [67]. PiC acts as a Pi/H^+^-symporter (influx of Pi and H^+^) or Pi/OH^−^-antiporter (influx of Pi, with efflux of OH^−^), and also as an exchange route for matrix/cytosolic Pi [68]. Uncoupling proteins (UCPs) are located in mitochondrial inner membranes, and dissipate the proton gradient across the inner mitochondrial membrane without ATP synthesis, thereby affecting oxidative phosphorylation in plants [69]. Another transporter, namely the dicarboxylate transport protein (DTP), catalyzes the exchange of Pi and dicarboxylate compounds like malate, succinate and malonate, and can also influence Pi concentrations in mitochondria in peas (*Pisum sativum*) [68]. There are also mitochondrial membrane-localized Pi transporters (MPT) that are localized in the mitochondrial inner membrane, and function in the Pi/H^+^-symport into the mitochondrial matrix. Among the three members of the *Arabidopsis* AtMPT family, the functions of AtMPT2 and AtMPT3 in Pi transport are known. *AtMPT3* overexpression in *Arabidopsis* alters plant growth by increasing ATP and reactive oxygen species (ROS) levels, and by boosting respiration rate [70]. Furthermore, PHT3 family members can also execute Pi transport into the mitochondria [57]. In *Arabidopsis*, three members of the *AtPHT3* family, namely *AtPHT3;1, AtPHT3;2*, and *AtPHT3;3*, are expressed in stems, leaves, and flowers, and are assumed to be involved in Pi import into mitochondria [54,57] (Figure 2). Liu et al. [57] performed homology-based analysis of *Arabidopsis* and rice *phosphate transporter* (*PT*) genes, and identified *OsPT15*, *16*, *17*, *18*, *19*, and *20* genes in a group with *Arabidopsis AtPHT3* genes (Figure 2). However, these rice OsPT proteins are localized in other organelles rather than the mitochondria [57]. Specifically, OsPT15, 17, 18, and 19 are localized in peroxisomes, OsPT16 in the endoplasmic reticulum (ER), and OsPT17 in the PM [57], which, together with the findings above, may suggest the functional divergence of these PT proteins in rice and *Arabidopsis* [54,57]. As such, mitochondrial Pi transporters of the PT family in rice are yet to be identified.

### 3.4. Cellular Movement of Pi Transporters Involves the ER

Phosphate transporter traffic facilitator 1 (PHF1) is a major regulator controlling the exit and movement of Pi transporters from the ER to the PM [71]. Ruan et al. [72] were able to modify the promoter of the rice *transporter traffic facilitator 1* (*OsPHF1*), a major factor regulating the ER-exit of Pi transporters to the PM, by using the *cis*-regulator high-affinity phosphate starvation response 1 binding sequence (HA-P1BS). The rice *OsPHF1-HA-P1BS* transgenic lines exhibited moderate upregulation of the *OsPHF1* gene, and improved Pi uptake efficiency under Pi-starvation conditions without adverse effects on growth in comparison with the transgenic plants constitutively overexpressing *OsPHF1* that displayed Pi overaccumulation in the shoots, and necrosis in the leaves [72]. In *Arabidopsis*, all *PHT1* genes also contain the P1BS *cis*-regulatory element in their promoter, whereas in rice, except for *OsPHT1;1* and *OsPHT1;4*, other *PHT1* members have the *P1BS cis*-element in their promoters. Additionally, *OsPHT1;2* was characterized as a direct target of OsPHR2 [73]. Transgenic rice plants overexpressing *OsPHF1* show remarkably higher Pi concentrations in shoots under both Pi-deficient and Pi-sufficient conditions than WT plants [74].

The trafficking of Pi transporters from the ER to the PM is regulated by phosphorylation mechanisms. A rice kinase subunit, casein kinase 2β3 (CK2β3), was found to interact with OsPT2 and OsPT8, and the holoenzyme, CK2α3/β3, was able to phosphorylate OsPT8 under Pi-sufficient conditions [75]. The phosphorylated OsPT8 could not interact with PHF1 for its movement from the ER to the PM under sufficiently Pi-supplied conditions; however, under Pi-starvation conditions, CK2β3 is degraded, and OsPT8 and PHF1 are able to interact. Therefore, under Pi-sufficient conditions, CK2α3/β3 acts as a negative regulator of PTs through the phosphorylation of PTs, thereby inhibiting the PHF1-mediated PT trafficking to the PM [75].

Endosomal complex required for transport (ESCRT) protein complexes assist in the trafficking of cargo proteins coming from the Golgi apparatus or the PM through intraluminal vesicles (ILV) of multivesiclar bodies (MVBs), prior to their release into the vacuolar lumen [76]. In a recent study, the cytosolic apoptosis-linked gene 2-interacting protein X (ALIX) was found to be associated with MVBs through the ESCRT-III subunit, sucrose non fermenting protein 7 (SNF7), for the trafficking of AtPHT1;1 into the vacuoles in *Arabidopsis*. The *Arabidopsis* *alix-1* mutants showed reduced trafficking of AtPHT1;1 into vacuoles for degradation, and enhanced Pi-starvation responses [76]. Correct ER-to-PM trafficking of AtPHT1;1 protein requires the function of AtPHF1 [71,77]. Under sufficiently Pi-supplied conditions, a CK holoenzyme phosphorylates AtPHT1;1, inhibiting its exit from the ER [76]. Alternatively, AtPHT1;1 protein is sent to vacuoles for degradation to decrease its levels at the PM, resulting in reduced Pi uptake under Pi-sufficient conditions [76]. Therefore, the involvement of the ER is crucial in maintaining the optimum concentration of PHTs on the PM in *Arabidopsis*.

### 3.5. Golgi Bodies

The Golgi body engages in several important reactions in plant cells, including the trafficking of proteins, and carbohydrate synthesis. The Golgi compartment hosts several ATP- and nucleotide sugar-dependent reactions that lead to the net release of Pi [78]. This released Pi needs to be exported back to the cytoplasm for the maintenance of Pi homeostasis. However, there is a huge knowledge gap on the Pi transport system in Golgi bodies. Subcellular localization studies in *Arabidopsis* indicated that only one member of the AtPHT4 family (i.e., AtPHT4;6) was targeted to the Golgi apparatus [52] (Figure 2). Mutation of this transporter led to inhibited growth and changes in the composition of the cell wall [58]. The reduction in AtPHT4;6 activity resulted in changes in Pi intracellular movement, and Golgi-mediated processes such as protein glycosylation [58]. AtPHT4;6 is responsible for the transport of Pi from Golgi bodies toward the cytosol [79] (Table 3). Transgenic *Arabidopsis atpht4;6* plants exhibited dark-induced senescence, suggesting that AtPHT4;6 regulates the critical function of controlling cellular Pi levels for the proper functioning of primary metabolism [59] (Figure 2).

## 4. Conclusions and Future Research Directions

The identification and characterization of various Pi transport proteins led to a significant accumulation of knowledge about Pi uptake and movement in plants. There are still major gaps in understanding Pi localization and transport activities among subcellular organelles within a plant cell under the conditions of both Pi-sufficient and Pi-deficient conditions. Research aiming to clarify how Pi transport activities are coordinated among various subcellular organelles, as well as cell types, under various developmental and environmental conditions is a future task, and requires a substantial effort from the research community. Despite the crucial roles of SPX-MFS proteins as vacuolar Pi transporters in buffering the cytoplasmic-Pi concentration against variable Pi availability, the molecular identity of the regulatory mechanisms via which Pi is translocated across the tonoplast remains elusive. Therefore, identification of other proteins involved in the efflux and influx of vacuolar Pi is needed. Additionally, the role of autophagy as an alternative cellular mechanism in recycling Pi through the delivery of RNA into vacuoles requires more attention. Although Pi is released from the RNA degradation in vacuoles, the contribution of such a Pi pool to the cytoplasmic Pi metabolism, and its biological relevance remain to be elucidated. To better understand the dynamics of cellular Pi homeostasis in a physiological context, we need new non-destructive quantitative methods with high detection sensitivity for measuring/visualizing Pi and relevant Pi-compounds, and their content in various subcellular compartments. With the recent development in next-generation sequencing technologies, several Pi deficiency-responsive micro RNA (miRNA) members have been identified in various plant species, but their roles in Pi uptake and transport are still far from being comprehensive. Furthermore, future research is also needed to discover the precise inter-relationships between Pi and other mineral (major and minor) elements. The roles of other important players, like redox and hormone homeostasis, in the regulation of Pi uptake and transport also need to be delineated. The efficiency of Pi use within a plant system needs to be examined in depth, which would ultimately enable us to enhance nutritional values and the adaptation potential of crops to meet the food demand of the ever-increasing population. This would also decrease the dependency on fertilizers, and would help in sustaining agricultural yields in the near future.

## Figures and Tables

**Table 1:** pH and membrane potential (Δψ) values in some plant species [e.g., *Arabidopsis*, rice (*Oryza sativa*), and spinach (*Spinacia oleracea*)] at the subcellular level (modified from Versaw and Garcia [15]).

Subcellular Organelle		pH	Δψ (Membrane Potential)	References
Mitochondrion		8.1	From −90 to −120 mV	[17,18]
Vacuole		5.2	+31 mV	[17,19]
Golgi body		6.3	Not known in plants	[17,20]
Plastid (non-photosynthetic)		7.3	−144 mV	[21]
Photosynthetic plastid	Thylakoid lumen	5.8–6.5	+30 mV	[22]
	Chloroplast stroma	8.0	−123 mV	[17,21]
Cytosol		7.3	−172 mV	[17]

**Table 2:** List of transporters involved in root uptake, root-to-shoot translocation, and redistribution and remobilization of phosphate in *Arabidopsis* (At) and rice (*Oryza sativa*, Os). PHO, Phosphate transporter; PHT, high-affinity phosphate transporter; VPT, vacuolar phosphate transporter.

Transporter(s)	Function	References
**Root Uptake**		
AtPHT1;1, AtPHT1;2, AtPHT1;3, AtPHT1;4	Involved in Pi uptake	[9,24,25,26]
OsPHT1;1, OsPHT1;2, OsPHT1;4, OsPHT1;6, OsPHT1;9, OsPHT1;10, OsPHT1;11, OsPHT1;13	Involved in Pi uptake/translocation. OsPHT1;11 and OsPHT1;13 play roles in Pi uptake in symbiotic association with arbuscular mycorrhizal fungi.	[27,28,29,30]
**Root-to-Shoot Translocation**		
AtPHT1;8, AtPHT1;9, AtPHO1, AtPHO1;H1, AtPHO1;H3	Translocation of Pi from roots to shoots. AtPHO1;H3 is involved in the suppression of root-to-shoot Pi transport under Zn-deficient conditions.	[9,31,32,33]
OsPHT1;2, OsPHT1;4, OsPHT1;6, OsPHT1;8, OsPHO1;2	Translocation of Pi from roots to shoots.	[34,35]
**Pi Redistribution and Remobilization**		
AtPHT1;1, AtPHT1;5, AtPHT1;9	AtPHT1;5 plays a role in Pi translocation from source to sink organs. Pi redistribution across the vegetative organs.	[24,36,37,38]
OsPHT1;4, OsPHT1;6, OsPHT1;8	OsPHT1;4 is involved in the remobilization of Pi from flag leaves to the panicles. OsPHT1;6 and OsPHT1;8 help in Pi remobilization from senescing leaves to young leaves and rice grains.	[3]

**Table 3:** List of transporters involved in phosphate transport in *Arabidopsis* and rice (*Oryza sativa*) at the subcellular level. ANTR1, putative anion transporter 1 (thylakoid Na^+^-dependent phosphate transporter); PHO, phosphate transporter; PHT, high-affinity phosphate transporter; PT, phosphate transporter; SPX-MFS3, SPX major facilitator superfamily 3.

Transporter(s)	Function	References
**Vacuole**		
OsSPXMFS1, AtVPT1/AtPHT5;1	Import	[1,19,48]
OsSPXMFS1 OsSPXMFS3	Import Export and symport	[19,48]
**Chloroplast**		
AtPHT2;1, AtPHT4;1, ANTR1 (leaf chloroplast) AtPHT4;2 (root plastid) AtPHT4;3 (shoot plastid) AtPHT4;4 (leaf chloroplast) AtPHT4;5 (shoot plastid)	Import and symport	[51,52,53]
**Mitochondrion**		
AtPHT3;1, AtPHT3;2, AtPHT3;3	Import and symport	[54,55,56]
OsPT15 (located on peroxisome) OsPT16 (located on endoplasmic reticulum) OsPT17 (located on peroxisome) OsPT18 (located on peroxisome) OsPT19 (located on peroxisome) OsPT20 (located on plasma membrane)	Import and symport	[57]
**Golgi Body**		
AtPHT4;6	Export	[58,59]

## Abbreviations

CK2β3	Casein kinase 2β3
ESCRT	Endosomal complex required for transport
GPT	Glucose-6-phosphate/phosphate translocator
MPT	Membrane-localized phosphate transporter
NLA	Nitrogen limitation adaptation
SNF	Sucrose non-fermenting protein
SPX-MFS	SYG1/PHO81/XPR major facilitator superfamily
P	Phosphorus
PHO	Phosphate transporter
PHF	Phosphate transporter traffic facilitator
PHT	High-affinity phosphate transporter
Pi	Phosphate
PPT	Phosphoenolpyruvate/phosphate translocator
pPT	Plastidic phosphate translocator
XPT	Xylulose-5-phosphate/phosphate translocator
VPT	Vacuolar phosphate transporter
